# Prognostic value of baseline EORTC QLQ-C30 scores for overall survival across 46 clinical trials covering 17 cancer types: a validation study

**DOI:** 10.1016/j.eclinm.2025.103153

**Published:** 2025-03-21

**Authors:** Luigi Lim, Abigirl Machingura, Mekdes Taye, Madeline Pe, Corneel Coens, Francesca Martinelli, Ahu Alanya, Stéphanie Antunes, Dongsheng Tu, Ethan Basch, Jolie Ringash, Yvonne Brandberg, Mogens Groenvold, Alexander Eggermont, Fatima Cardoso, Jan Van Meerbeeck, Michael Koller, Winette T.A. Van der Graaf, Martin J.B. Taphoorn, Johan A.F. Koekkoek, Jaap C. Reijneveld, Riccardo Soffietti, Galina Velikova, Andrew Bottomley, Henning Flechtner, Jammbe Musoro, EORTC Quality of Life Group, Brain Tumour Group, Breast Cancer Group, Melanoma Group, Lung Cancer Group, Soft Tissue and Bone Sarcoma Group, Radiation Oncology Group, Lymphoma Group, Gastrointestinal Tract Cancer Group, Head and Neck Cancer Group, Genito-Urinary Cancer Group, Gynaecological Cancer Group

**Affiliations:** a European Organisation for Research and Treatment of Cancer (EORTC), Brussels, Belgium; b Formerly European Organisation for Research and Treatment of Cancer (EORTC) HQ, Brussels, Belgium; c Princess Margaret Cancer Centre and the University of Toronto, Canada; d Canadian Cancer Trials Group, Queen's University, Canada; e Department of Child and Adolescent Psychiatry, Otto-von-Guericke-University Magdeburg, Magdeburg, Germany; f Department of Oncology-Pathology, Karolinska Institutet, Sweden; g Department of Public Health, University of Copenhagen and Palliative Care Research Unit, Bispebjerg/Frederiksberg Hospital, Denmark; h Princess Máxima Center, Utrecht and University Medical Center Utrecht, the Netherlands; i Comprehensive Cancer Center Munich of the Technical University Munich and the Ludwig Maximiliaan University, Munich, Germany; j Breast Unit, Champalimaud Clinical Center/Champalimaud Foundation, Lisbon, Portugal; k Antwerp University and Antwerp University Hospital, Edegem, Belgium; l Department of Medical Oncology, Netherlands Cancer Institute Amsterdam, the Netherlands; m Department of Medical Oncology Erasmus MC Cancer Institute, Erasmus MC, Rotterdam, the Netherlands; n Lineberger Comprehensive Cancer Center, UNC, USA; o Leiden University Medical Center and Haaglanden Medical Center, The Hague, the Netherlands; p Azienda Ospedaliera, Universita di Torino, Italy; q Department of Neurology, Amsterdam University Medical Centre, Amsterdam, Netherlands & Sein, Heemstede, the Netherlands; r Leeds Institute of Medical Research at St James's University, University of Leeds and Leeds Cancer Centre, Leeds Teaching Hospitals NHS Trust, St James’s University Hospital, Leeds, UK; s Center for Clinical Studies, University Hospital Regensburg, Regensburg, Germany

**Keywords:** Cancer clinical trial, Health-related quality of life, Survival, Prognostic factor analysis, Validation, Pooled data

## Abstract

**Background:**

A pooled data analysis by Quinten et al. (2009) found three European Organisation for Research and Treatment of Cancer Quality of Life Questionnaire Core 30 (EORTC QLQ-C30) health-related quality of life (HRQoL) scales to be prognostic for survival: physical functioning, pain and appetite loss. This study aims to replicate these findings in an independent data set comprising a broader cancer population.

**Methods:**

Data were obtained from 46 clinical trials across three cancer research networks conducted between 1996 and 2013 that assessed HRQoL using the EORTC QLQ-C30. A stratified Cox proportional hazards model was employed to assess the prognostic significance of baseline QLQ-C30 scale scores on overall survival, adjusting for socio-demographic and clinical variables. Stepwise model selection was done at 5% significance level. Model stability and prognostic accuracy were evaluated via bootstrapping and the *C* index respectively.

**Findings:**

Data from 16,210 patients reporting HRQoL at baseline, spanning 17 cancer types, was used. The stratified multivariable model confirmed that better physical functioning (hazard ratio [HR], 0.94; 95% confidence interval [CI], 0.93–0.96), lower pain (HR, 1.02; 95% CI, 1.01–1.03), and appetite loss (HR, 1.04; 95% CI, 1.03–1.05) were significantly associated with survival. Additionally, global health status/QoL, dyspnoea, emotional and cognitive functioning were found to be prognostic for survival. This final model, encompassing sociodemographic, clinical, and HRQoL variables, achieved a corrected *C* index of 0.74, marking a 48% enhancement in discriminatory ability. Bootstrap evaluation indicated no major instability issues.

**Interpretation:**

These results support previous findings that baseline physical functioning, pain, and appetite loss scores, along with four other scales from the EORTC QLQ-C30, predict survival in cancer patients.

**Funding:**

EORTC Quality of Life Group.

**Research in context**

**Evidence before this study**

The growing importance of health-related quality of life (HRQoL) in cancer clinical research has led to increase integration of HRQoL into clinical trials, spurring further research into the relationship between HRQoL and survival. The prognostic value of HRQoL, as measured by the European Organisation for Research and Treatment of Cancer Quality of Life Questionnaire Core 30 (EORTC QLQ-C30), on survival has been explored in numerous cancer types which have identified several prognostic scales. A 2009 series in *The Lancet Oncology* by Quinten et al. found three EORTC QLQ-C30 scales (appetite loss, pain, and physical functioning) to be prognostic for survival in a pooled analysis of 7417 patients across 11 cancer types. However, these findings were all exploratory and replication is needed to ensure their robustness and generalizability. A search on PubMed, from database inception to June 25, 2024, for papers published in English, using the terms “prognostic factor analysis”, “EORTC QLQ-C30”, “HRQoL”, “cancer”, “clinical trials”, “pooled data” and “validation” yielded 3 results. Additionally, studies were found by reviewing the references of identified publications. Studies were limited in terms of cancer type and research networks considered in the population.

**Added value of this study**

This study validated the findings from Quinten et al. in an independent dataset, encompassing data from 16,210 patients across 17 cancer types, coordinated by different research networks for enhanced generalizability. Our results confirm that baseline scores for physical functioning, pain, appetite loss, and four other EORTC QLQ-C30 scales can predict survival in cancer patients. However, imposing a uniform set of prognostic factors across cancer types may dampen the impact of HRQoL, as its prognostic significance can vary by cancer type.

**Implications of all the available evidence**

This study underscores the prognostic value of HRQoL, important for both clinical trial design and clinical practice. While using baseline HRQoL scores as stratification factors in trials can improve efficiency and reduce confounding, challenges with multiple prognostic scales highlight the need for further guidance. Additionally, integrating HRQoL assessments into practice can help clinicians prioritise key scales to enhance prognosis and address significant impairments.

## Introduction

The use of health-related quality of life (HRQoL), alongside other patient-reported outcomes (PROs), has become more common in randomised cancer clinical trials. This trend could be attributed to several factors. Symptoms or loss in functioning may be best reported by the patient (e.g., pain or fatigue).^1^ HRQoL provides additional valuable insights into the burden of cancer and treatment effectiveness from the patient's perspective, aiding decision-making for clinicians and patients.^2^ Furthermore, regulatory bodies such as the US Food and Drug Administration (FDA) and the European Medicines Agency (EMA) have noted the added value of HRQoL to standard efficacy and safety outcomes and have released guidelines on the use of PROs in the drug evaluation process.^3^, ^4^, ^5^, ^6^ All these, among others, have contributed to the more widespread integration of HRQoL in clinical trials.

Apart from being recognised as a relevant endpoint in cancer trials,^7^ there has also been growing interest in studying how HRQoL of cancer patients could be predictive of their survival. Research has explored which aspects of HRQoL are prognostic for survival across various cancers, including lung,^8^, ^9^, ^10^, ^11^ breast,^12^, ^13^, ^14^, ^15^, ^16^, ^17^ brain,^18^^,^^19^ liver,^20^ and bladder,^21^ among others. Most of these analyses show consistent relationships between HRQoL and survival. Gotay et al.^22^ found that 36 out of the 39 studies assessed had at least one HRQoL variable significantly associated with survival, although effect sizes and identified scales varied across studies. An updated review by Mierzynska et al.^23^ which assessed more recent studies, when recommendations were available about optimizing methodological rigour^24^ of HRQoL prognostic factor analyses, had similar findings (41 out of 44 studies). Establishing HRQoL as a prognostic factor could benefit clinical trial design as an inclusion criterion or stratification factor^25^ to create (sub)populations with homogeneous overall survival (OS).

Previously, Quinten et al.^25^ performed a pooled analysis of 7417 patients to look at the association between survival and HRQoL as measured by the European Organisation for Research and Treatment of Cancer Quality of Life Core Questionnaire (EORTC QLQ-C30) across different cancer types. They found that physical functioning, pain, and appetite loss were prognostic for survival. The addition of these scales was also found to improve the prognostic accuracy by 6% compared to a model composed of clinical and sociodemographic variables only. However, their study was limited to certain cancer types and trials conducted by EORTC.

Although these studies already show the prognostic value of HRQoL, the conclusions on the generalizability of HRQoL as prognostic of overall survival remain limited since these analyses were largely exploratory and focused on specific cancer types. Replicating these findings with a pre-defined hypothesis is necessary to strengthen their robustness and facilitate the integration of these prognostic models into clinical practice.^26^ Although a previous validation study^27^ of Quinten et al.^25^ had been done, the type of cancers considered in that study were similarly limited. Based on the study by Quinten et al.,^25^ we hypothesise that poor baseline scores in self-reported physical functioning, pain, and appetite loss are consistently associated with worse overall survival across all QLQ-C30 scales. The present analysis aims to cross-validate and extend the prognostic value of these scales on overall survival in an independent dataset from clinical trials across different cancer types, coordinated by different research organisations, and to evaluate if this applies to a broader cancer population.

## Methods

### Ethics

This research project was checked by The Ethics Committee Hospitalo-Facultaire Saint-Luc UCL (ethics approval number: 2019/29AOU/375). The use of the patient data from the various studies fell under their original informed consent wording, hence, no additional consent was needed. The original studies were conducted in compliance with the Declaration of Helsinki.

### Study selection

This study used 46 closed randomised controlled trials (RCTs) with HRQoL assessments at baseline conducted by the European Organisation for Research and Treatment of Cancer (EORTC), the Mayo Clinic, or the Canadian Cancer Trials Group (CCTG) between 1996 and 2013. The period considered was based on the availability of published trial data eligible for data sharing at the time of the grant application. Baseline assessments were taken within 2 weeks before or after randomisation, but before treatment start. Trials used in the prognostic factor analysis of Quinten et al.^25^ were excluded from the selection.

### Data collection

HRQoL was assessed using EORTC QLQ-C30^28^^,^^29^ (version 3), a widely used quality of life questionnaire for cancer patients.^30^ It includes 9 multi-item scales i.e., five functional scales: physical, role, emotional, cognitive, and social functioning, three symptom scales: fatigue, pain, and nausea and vomiting, and a global health status/QoL scale. Six single-item scales assess symptoms: dyspnoea, appetite loss, sleep disturbance, diarrhoea, and financial impact.^28^ Scale scores are transformed to a 0–100 range, with higher scores indicating better functioning for functional scales, and greater symptom burden for symptom scales.^29^

The following clinical and sociodemographic characteristics known to be prognostic to patient survival were also collected: sex, age, WHO performance status (PS), and metastasis status at study entry. Similar to Quinten et al.,^25^ age was dichotomized as ≤60 years and >60 years. Due to imbalance in PS score distribution, patients' scores were dichotomized into active (PS = 0) and restricted (PS = 1 to 4).

### Statistical analysis

The Cox proportional hazards model (CPHM)^31^ was used to assess the prognostic value for baseline QLQ-C30 scale scores on overall survival (OS); measured from the time of randomisation until death (due to any cause) or censored at time last known alive. All CPHMs were stratified by cancer type and organisation conducting the trial (i.e., EORTC, CCTG, and Mayo Clinic) to address violations of the proportional hazards assumption. Trials were not used as a stratification factor as this led to more and smaller strata. The model building procedure was implemented in the following 3 steps:

Step 1 Spearman rank correlations (ρ) between all explanatory variables were computed to screen pairs of highly correlated variables (i.e., |ρ| ≥ 0.80). Univariable CPHMs were fitted for each sociodemographic, clinical, and HRQoL variable to inform the multivariable analyses (*step 2*).
Step 2 A multivariable CPHM was fitted with the retained variables from *step 1* using a stepwise selection procedure, where the stay and entry criteria were both set at a p-value threshold of 0.05. To evaluate the added prognostic value of baseline HRQoL scale scores, two multivariable CPHMs were considered. The first model, CPHM_X_, evaluated the prognostic value of sociodemographic and clinical variables alone. The second model (CPHM_QX_) included the HRQoL scales from *step 1*. To obtain the final models, two modelling strategies were used for the stepwise selection procedure. The first strategy (CPHM_QX1_) only retained sociodemographic, clinical and HRQoL variables with p-values ≤ 0.05. The second strategy (CPHM_QX2_) always retained all sociodemographic and clinical variables from *step 1* irrespective of p-value and only HRQoL variables with p-values ≤ 0.05. The assumption of proportional hazards was checked for the final models (CPHM_QX1_ and CPHM_QX2_) using Kaplan–Meier curves on the log-log scale.^32^ The prognostic value of each variable was assessed using the hazard ratio (HR), its 95% confidence interval, and the p-value of its Wald χ^2^ test statistic. For the HRQoL scales, the HRs were computed based on 10-point differences in the scores, approximating a “minimal” important difference for the EORTC QLQ-C30.^33^, ^34^, ^35^

Harrell’s *C* Index^36^ was used to assess the discrimination ability of the final models. The *C* index is an estimate of the probability of concordance between observed and predicted survival times, with *C* = 0.5 for a model indicating no predictive discrimination and *C* = 1 for perfect discrimination.

Step 3 A bootstrap procedure was implemented to evaluate the stability of selected predictors in the final models and correct for overfitting for the *C* index (details in Appendix).

Kaplan–Meier plots were used to demonstrate the prognostic ability of selected QLQ-C30 scale scores in a subset of patients with good prognosis based on sociodemographic and clinical variables retained in the final models. Additionally, the final CPHM from Quinten et al.^25^ was also fitted to our study data to obtain the apparent *C* index to compare to our corrected *C* index. Our results were also descriptively compared with those of Quinten et al.^25^ Post-hoc analyses were performed to assess the robustness of our findings by applying the model-building strategy to non-metastatic and metastatic cohorts separately. A forest plot of HRs for common scales from both Quinten et al. and our study, based on a multivariate model with clinical and sociodemographic variables, was also created to examine consistency across cancer types. All statistical analyses were performed using SAS 9.4 software.^37^

### Role of the funding source

The funder of the study had no role in study design, data collection, data analysis, data interpretation, or writing of the report.

## Results

### Descriptive results

Baseline data from 16,863 patients were pooled from 46 closed RCTs. Of these, 624 (4%) patients were excluded because of a missing baseline HRQoL scale score, 29 (0.2%) because of missing survival data, and 2901 (17%) because of missing sociodemographic data (See Appendix Fig. A2). The analysis data set for the Cox models consisted of 13,309 patients across 17 cancer types with complete baseline and survival data. The current study included seven additional cancer types compared to the previous studies, namely: bladder, gastric, endometrial, and anal cancers, soft tissue sarcoma, lymphoma, and multiple myeloma. Oesophageal cancer, which represented 0.8% of the sample in Quinten et al.,^25^ was the only cancer not represented in this study.

Table 1 shows the distribution of baseline characteristics for the patients included in the analysis. Ages ranged from 16 to 93 years, with a median age of 58 years. Over half of the patients had non-metastatic disease (54%) and the most common cancer type was breast (21%). Patients were nearly balanced between active (45%) and restricted (46%) PS. Patients had a median survival time of 32.8 months (95% CI: 31.2–34.5).

**Table 1:** Patient baseline characteristics.

Patient Characteristic	Total (N = 16,210)
	N (%)
**Age**	
Median	58.0
Range	16–93
≤60 years	9130 (56.3)
>60 years	7080 (43.7)
**Sex**	
Male	6647 (41.0)
Female	9563 (59.0)
**Cancer type**	
Lung	2732 (16.9)
Melanoma	861 (5.3)
Lymphoma	348 (2.1)
Testicular	270 (1.7)
Prostate	645 (4.0)
Breast	3466 (21.4)
Brain	822 (5.1)
Bladder	228 (1.4)
Gastric	324 (2.0)
Pancreatic	516 (3.2)
Ovarian	2269 (14.0)
Endometrial	92 (0.6)
Soft Tissue sarcoma	425 (2.6)
Colorectal	1693 (10.4)
Anal	60 (0.4)
Head and Neck	330 (2.0)
Multiple myeloma	318 (2.0)
Multiple types	811 (5.0)
**WHO performance status**	
Active (Performance status = 0)	7211 (44.5)
Restricted	7558 (46.6)
Performance status = 1	6317 (39.0)
Performance status = 2	1157 (7.1)
Performance status = 3	84 (0.5)
Missing	1441 (8.9)
**Metastasis status**	
Metastatic	5709 (35.2)
Non-metastatic	8837 (54.6)
Missing	1664 (10.2)

The average baseline QLQ-C30 scales scores for the total sample, by different sociodemographic and clinical characteristics, are shown in Table 2. On average, patients had a global health status/QoL score of 65 (SD = 23). Role functioning was the most impaired functional scale with an average score of 71 (SD = 32) while fatigue was the most impaired symptom scale with an average score of 33 (SD = 26). Patients under 60 years old generally had better functioning and fewer severe symptoms than those over 60 except for a few select scales (emotional functioning, insomnia, pain, and financial problem scales). Male and female patients had similar scores across most HRQoL scales. Fully active patients (PS 0) had markedly higher functional scale scores and lower symptom scale scores, showing the starkest difference among all sociodemographic and clinical characteristics. These higher scores were also seen when comparing metastatic and non-metastatic patients albeit to a lesser extent.

**Table 2:** Mean and Standard Deviation of baseline HRQoL scale scores of the whole patient population and by patient characteristics at baseline (N = 16,210).

HRQoL Scale	Overall	Age Group		Sex		WHO Performance Status^a^		Metastasis Status	
	All	≤ 60	>60	Male	Female	Active	Restricted	Metastatic	Non-metastatic
**All observations**	**16,210**	**9130**	**7080**	**6647**	**9563**	**7211**	**7558**	**5709**	**8837**
Physical functioning	80 (21)	83 (20)	76 (22)	79 (22)	80 (21)	88 (16)^b^	70 (23)	75 (22)	84 (19)
Role functioning	71 (32)	72 (31)	70 (32)	71 (32)	72 (31)	81 (26)^b^	59 (33)	67 (32)	76 (30)
Emotional functioning	73 (22)	71 (22)	75 (22)	76 (22)	70 (23)	76 (21)	70 (23)	73 (22)	73 (22)
Cognitive functioning	84 (21)	84 (20)	83 (21)	85 (20)	83 (21)	87 (18)	80 (22)	85 (20)	85 (20)
Social functioning	76 (28)	76 (28)	76 (28)	76 (27)	75 (28)	82 (24)^b^	66 (30)	72 (29)	79 (26)
Global health status/QoL	65 (23)	67 (23)	62 (23)	63 (23)	66 (23)	73 (20)^b^	55 (23)	60 (23)	69 (22)
Fatigue	33 (26)	32 (26)	35 (26)	33 (26)	33 (26)	24 (22)^b^	44 (26)	38 (26)^b^	28 (25)
Nausea/vomiting	15 (20)	14 (19)	17 (20)	14 (19)	16 (20)	9 (15)^b^	23 (22)	19 (20)	11 (18)
Pain	26 (28)	26 (28)	25 (28)	26 (29)	25 (28)	18 (23)^b^	35 (31)	31 (29)^b^	20 (25)
Dyspnoea	18 (26)	16 (25)	21 (27)	21 (27)	16 (25)	11 (21)^b^	26 (29)	25 (28)^b^	13 (23)
Insomnia	30 (31)	31 (31)	27 (30)	27 (30)	32 (31)	26 (29)	34 (32)	31 (31)	28 (30)
Appetite loss	20 (29)	18 (28)	22 (31)	20 (29)	20 (29)	12 (23)^b^	29 (33)	26 (31)^b^	15 (26)
Constipation	15 (26)	14 (25)	18 (28)	15 (26)	16 (27)	11 (22)^b^	21 (30)	19 (28)	12 (24)
Diarrhoea	8 (18)	8 (18)	8 (19)	8 (18)	8 (19)	7 (17)	9 (20)	9 (19)	7 (17)
Financial problems	17 (28)	21 (31)^b^	11 (23)	18 (29)	16 (28)	15 (26)	21 (30)	19 (29)	15 (27)

a Active WHO Performance Status (PS = 0); Restricted WHO Performance Status (PS = 1 to 4).

b Differences that are clinically meaningful based on a 10-pt. threshold.

Average HRQoL scale scores across the different cancer types are shown in Table 3. Pancreatic and ovarian cancer patients had worse average scores than the total patient population. Melanoma and prostate cancer patients, on the other hand, reported better than average quality of life across all scales. Different symptoms impacted cancer types differently. For example, worse scores obtained for appetite loss for pancreatic cancer, dyspnoea for lung cancer, constipation for bladder cancer, and diarrhoea for gastric cancer.

**Table 3:** Mean and Standard Deviation of baseline HRQoL scale scores of the whole patient population and by disease site at baseline (N = 16,210).

HRQoL Scale	Overall	Anal	Bladder	Brain	Breast	Colorectal	Endometrial	Gastric	Head and Neck	Lung
**All observations**	**16,210**	**60**	**228**	**822**	**3466**	**1693**	**92**	**324**	**330**	**2732**
Physical functioning	80 (21)	82 (20)	70 (26)	79 (23)	90 (15)	79 (19)	75 (23)	87 (16)	87 (17)	72 (21)
Role functioning	71 (32)	74 (29)	57 (37)	65 (33)	85 (24)	74 (28)	64 (33)	77 (28)	82 (26)	67 (31)
Emotional functioning	73 (22)	63 (24)	65 (27)	73 (23)	70 (21)	79 (19)	68 (24)	72 (23)	73 (23)	74 (23)
Cognitive functioning	84 (21)	78 (25)	80 (25)	70 (27)	85 (19)	87 (17)	83 (21)	85 (20)	89 (17)	84 (20)
Social functioning	76 (28)	78 (26)	69 (33)	68 (30)	84 (23)	74 (27)	76 (28)	77 (28)	86 (21)	73 (27)
Global health status/QoL	65 (23)	63 (23)	50 (25)	64 (23)	74 (21)	63 (22)	64 (23)	66 (23)	62 (22)	60 (22)
Fatigue	33 (26)	33 (26)	46 (31)	35 (25)	23 (21)	35 (24)	35 (27)	33 (27)	26 (25)	39 (25)
Nausea/vomiting	15 (20)	5 (14)	12 (22)	14 (18)	10 (16)	19 (18)	10 (18)	11 (19)	4 (13)	22 (20)
Pain	26 (28)	33 (32)	41 (36)	13 (21)	18 (23)	27 (27)	30 (30)	27 (27)	28 (25)	27 (28)
Dyspnoea	18 (26)	12 (24)	19 (27)	11 (21)	9 (19)	21 (25)	14 (26)	15 (24)	13 (22)	32 (30)
Insomnia	30 (31)	39 (33)	39 (37)	26 (32)	31 (29)	26 (28)	35 (32)	28 (30)	27 (30)	27 (30)
Appetite loss	20 (29)	18 (26)	36 (38)	10 (22)	11 (21)	25 (29)	28 (30)	19 (28)	22 (30)	25 (31)
Constipation	15 (26)	23 (33)	35 (36)	13 (25)	9 (19)	15 (25)	24 (33)	15 (25)	15 (26)	17 (27)
Diarrhoea	8 (18)	8 (15)	8 (20)	5 (15)	6 (15)	11 (21)	9 (20)	18 (28)	4 (13)	6 (16)
Financial problems	17 (28)	15 (24)	13 (26)	14 (27)	15 (27)	17 (27)	14 (26)	18 (29)	18 (28)	22 (31)

HRQoL scale	Lymphoma	Melanoma	Mixed population	Multiple myeloma	Ovarian	Pancreatic	Prostate	Soft tissue sarcoma	Testicular
**All observations**	**348**	**861**	**811**	**318**	**2269**	**516**	**645**	**425**	**270**
Physical functioning	78 (21)	92 (12)	60 (27)	78 (17)	75 (22)	75 (21)	90 (14)	77 (22)	82 (23)
Role functioning	57 (34)	84 (24)	50 (34)	69 (29)	59 (35)	61 (33)	91 (19)	71 (31)	57 (35)
Emotional functioning	67 (24)	82 (18)	68 (23)	82 (18)	69 (24)	67 (24)	83 (17)	74 (23)	67 (24)
Cognitive functioning	84 (21)	93 (14)	76 (24)	86 (17)	81 (22)	81 (22)	87 (16)	85 (20)	87 (19)
Social functioning	72 (30)	88 (20)	64 (31)	70 (27)	67 (31)	66 (30)	93 (15)	75 (28)	68 (31)
Global health status/QoL	55 (22)	78 (18)	53 (24)	67 (19)	59 (23)	54 (23)	77 (18)	64 (23)	58 (23)
Fatigue	49 (26)	15 (18)	47 (28)	33 (21)	41 (26)	45 (27)	15 (18)	33 (25)	40 (28)
Nausea/vomiting	10 (18)	1 (6)	34 (23)	22 (18)	17 (23)	21 (21)	2 (7)	7 (15)	12 (19)
Pain	34 (32)	12 (20)	56 (32)	30 (26)	30 (28)	38 (30)	10 (18)	29 (30)	44 (34)
Dyspnoea	30 (30)	5 (14)	25 (30)	14 (21)	19 (27)	17 (25)	12 (21)	20 (27)	17 (27)
Insomnia	49 (36)	18 (26)	39 (33)	25 (28)	35 (32)	34 (31)	16 (23)	26 (29)	38 (35)
Appetite loss	29 (33)	3 (12)	27 (33)	14 (24)	25 (33)	42 (35)	3 (11)	17 (27)	33 (33)
Constipation	16 (27)	5 (13)	24 (32)	7 (18)	23 (31)	27 (33)	7 (18)	15 (26)	17 (28)
Diarrhoea	9 (20)	6 (15)	8 (20)	7 (16)	10 (21)	15 (26)	6 (15)	7 (17)	8 (18)
Financial problems	23 (33)	14 (26)	20 (30)	23 (30)	14 (26)	21 (30)	3 (11)	19 (29)	23 (32)

Spearman rank correlations showed no strong relationship among sociodemographic, clinical, and HRQoL variables (i.e., |ρ| ≥ 0.8). Univariable Cox models (See Table 4) showed all candidate predictors significantly predicted survival, consistent with Quinten et al.^25^ Patients with metastatic disease had nearly double the risk of death until the end of follow-up. Those with restricted PS had an increased risk of death of 55.3%. Women and those ≤60 years old had better overall survival. All 15 QLQ-C30 scales significantly predicted overall survival. Complementary log-log plot of the survival curves indicated no violation of proportionality assumptions for the sociodemographic and clinical variables.

**Table 4:** Univariable and Multivariable Analysis of Clinical and HRQoL Variables and Results from Quinten et al. (2009).

	Univariable Cox Models	Cox Model for sociodemographic and clinical data (CPHM_X_)	Cox model for sociodemographic, clinical, and HRQoL data (CPHM_QX1_/CPHM_QX2_)	Our data analysed in the Cox Model of Quinten et al. (2009)	Results of Quinten et al. (2009) for comparison
	Hazard ratio (95% CI)	Hazard ratio (95% CI)	Hazard ratio (95% CI)	Hazard ratio (95% CI)	Hazard ratio (95% CI)
Sociodemographic and clinical variables^a^					
Sex (Female vs. Male)	0.867 (0.823, 0.913)	0.890 (0.841, 0.942)	0.869 (0.820, 0.919)	0.852 (0.805, 0.901)	0.74 (0.67–0.82)
Age (>60 vs. ≤ 60)	1.181 (1.126, 1.239)	1.117 (1.063, 1.174)	1.105 (1.051, 1.162)	1.115 (1.061, 1.172)	1.17 (1.06–1.28)
WHO PS (Restricted vs. Active)	1.553 (1.479, 1.630)	1.505 (1.429, 1.584)	1.239 (1.173, 1.308)	1.259 (1.192, 1.329)	1.07 (0.97–1.19)
Metastases (Yes vs. No)	1.900 (1.772, 2.038)	1.858 (1.733, 1.993)	1.800 (1.678, 1.930)	1.823 (1.700, 1.955)	1.70 (1.49–1.93)
QLQ-C30 HRQoL subscales^b^^,^^c^					
Physical functioning	0.882 (0.873, 0.891)		0.940 (0.926, 0.955)	0.932 (0.919, 0.945)	0.94 (0.92–0.96)
Pain	1.079 (1.071, 1.088)		1.023 (1.013, 1.034)	1.022 (1.011, 1.032)	1.04 (1.02–1.06)
Appetite loss	1.082 (1.075, 1.090)		1.042 (1.033, 1.052)	1.042 (1.034, 1.051)	1.05 (1.03–1.06)
Global health status/QoL	0.900 (0.892, 0.909)		0.964 (0.950, 0.977)		
Dyspnoea	1.064 (1.056, 1.073)		1.014 (1.004, 1.023)		
Emotional functioning	0.967 (0.958, 0.977)		1.025 (1.012, 1.037)		
Cognitive functioning	0.959 (0.949, 0.969)		1.034 (1.020, 1.048)		
Role functioning	0.927 (0.921, 0.934)				
Social functioning	0.940 (0.933, 0.947)				
Fatigue	1.097 (1.088, 1.106)				
Nausea/Vomiting	1.111 (1.099, 1.123)				
Insomnia	1.023 (1.016, 1.030)				
Constipation	1.039 (1.031, 1.047)				
Diarrhoea	1.013 (1.002, 1.024)				
Financial problems	1.021 (1.013, 1.028)				

a Reference categories are Female, >60, Restricted, and Yes.

b HRs are computed reflecting a 10-pt increase in the scale score.

c Higher functioning and lower symptom scale scores indicate better HRQoL.

### Main results

For CPHM_X_, all variables remained highly significant prognostic factors for survival. Both methods fitting HRQoL (CPHM_QX1_ and CPHM_QX2_) ended up selecting the same set of predictors. These two methods are compared further in the text. All sociodemographic or clinical variables and seven of the 15 QLQ-C30 scales were retained after the stepwise selection procedure. The second and third columns of Table 4 show the HRs for the three models. For most HRQoL scales, better scores were associated with better survival. All QLQ-C30 scales selected in Quinten et al.^25^’s model were also selected our final model: physical functioning, pain, and appetite loss. Additionally, our study also found global health status/QoL, dyspnoea, emotional functioning, and cognitive functioning scales to be prognostic (Table 4). However, unexpectedly, better emotional and cognitive functioning were associated with worse survival, contrary to the univariable analysis (Table 4, first column). The fourth column of Table 4 shows the estimates of the final model of Quinten et al.^25^ fitted on our study data. The HRs from this model closely match both the estimates from our final multivariable model (third column) and the original model by Quinten et al.^25^ (fifth column).

### Predictive ability

The corrected *C* index was used to estimate the difference in discriminatory ability between the two multivariable models. Corrections for overfitting were minimal, with the largest difference between naïve and corrected estimates being 0.0005. The model containing only sociodemographic and clinical variables had a corrected *C* index of 0.72 which is a 44% ((0.72–0.5)/0.5) improvement over a model with no discriminative ability. The model which includes sociodemographic, clinical, and HRQoL variables had a corrected *C* index of 0.74 representing a 48% ((0.74–0.5)/0.5) improvement in discriminatory ability. Differences in the corrected *C* indices between the two model building strategies were also minimal (0.00001). Thus, the addition of the seven significant QLQ-C30 scales resulted in a 2.8% ((0.74–0.72)/0.72) improvement in relative discriminatory ability. We obtained an apparent *C* index of 0.737 from the final model of Quinten et al.^25^ when applied to on the current data, a decrease of 0.003 compared to the corrected *C* index of our final multivariable model.

### Model stability

Appendix Fig. A3 displays the bootstrap procedure result, showing the selection proportion of covariates across both model building strategies. For both strategies, covariates retained in the final model were consistently selected in over 95% of the 200 bootstrap samples, except for the dyspnoea scale (64.5%). Dropped QLQ-C30 scales had selection proportions below 50%.

### Exploratory analyses

**Fig. 1:**
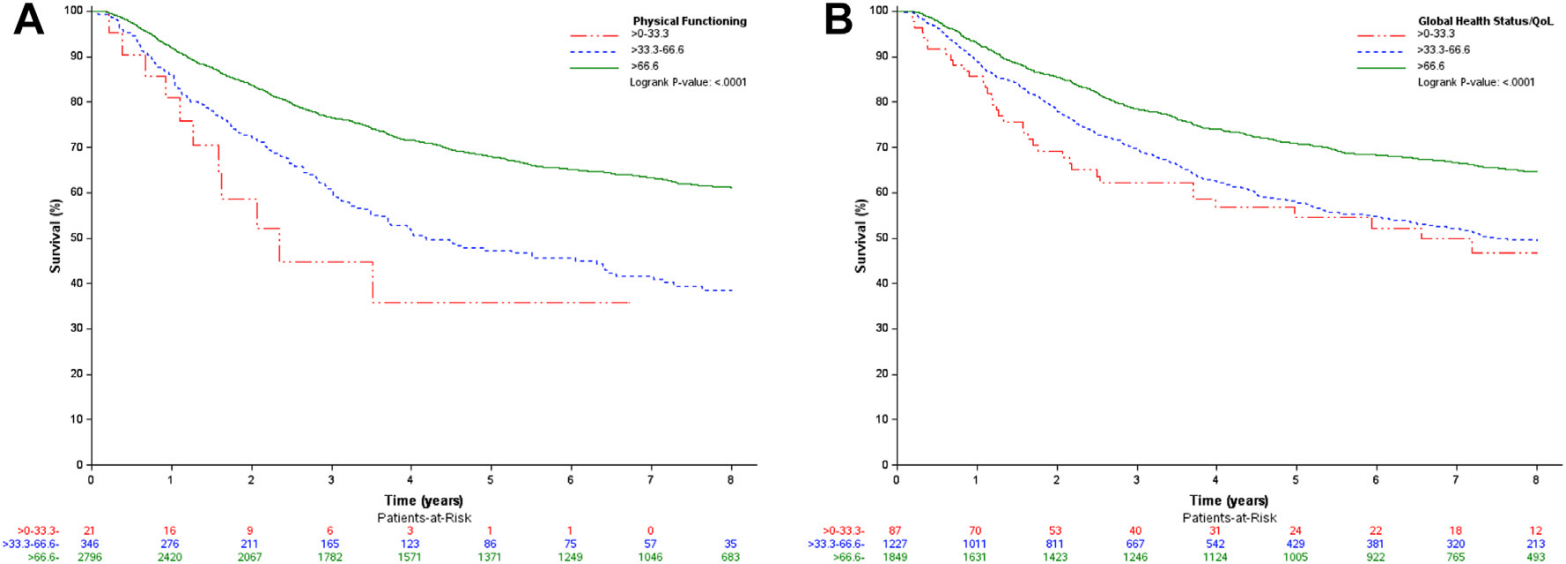
Kaplan–Meier Curves of patients with good prognosis by Physical Functioning (A) and Global Health Status/QoL (B). Physical functioning and Global health status/QoL scale scores were categorized to construct the Kaplan–Meier curves. Patients with good prognosis were defined as female, 60 or younger, with active WHO performance status (PS 0). Higher scores indicate better HRQoL.

To further demonstrate the added prognostic value of the retained QLQ-C30 scales beyond improvement in *C* index, Kaplan–Meier curves were constructed for patients with good prognosis. The profile of these patients was: females, 60 or younger, with active PS. Metastasis status was left out because this resulted in too small samples for the categorised scales. Fig. 1A and B shows the Kaplan–Meier curves for the categorised scores of physical functioning and global health status/QoL, respectively. The curves show clear separation in the expected direction with higher scores surviving longer. Other retained scales showed similar patterns except for emotional and cognitive functioning where moderate scores had better survival times (see Appendix).

### Post-hoc analyses

Most scales retained in the overall sample were also retained in the metastatic cohort, except for dyspnoea (excluded) and Fatigue and Nausea/Vomiting (included only in the metastatic cohort). In the non-metastatic cohort, only physical functioning and appetite loss were retained, both of which were also selected in the metastatic cohort and pooled sample cohorts (Appendix Table A3). Appendix Figs. A4–A6 present a forest plot of HRs for physical functioning, appetite loss, and pain scales (common to both Quinten et al. and our study) in the pooled sample, by metastasis cohorts and cancer type. The results show that HR magnitudes were similar across most cancer types and metastasis statuses, though not always significantly prognostic for survival.

## Discussion

This study validated previous research by Quinten et al.^25^ regarding the added prognostic value of HRQoL beyond clinical and sociodemographic factors. The validation was performed in a large, independent dataset of patients representing 17 different cancer types who were due to participated in a clinical trial. The inclusion of previously underrepresented or unrepresented cancer types showed that the additional prognostic value of HRQoL applies to patients in an even broader context than previously established. Like Quinten et al.,^25^ we found that baseline physical functioning, pain, and appetite loss were prognostic for OS. Additionally, our study uniquely identified baseline emotional functioning, cognitive functioning, global health status/QoL and dyspnoea as prognostic factors. Ediebah et al.^27^ also reported global health status/QoL and dyspnoea to be prognostic for survival in a pooled sample of Canadian cancer patients. Previous prognostic factor analyses also found emotional functioning^18^ and cognitive functioning^15^^,^^19^ to be prognostic for survival. Systematic reviews consistently found physical functioning and global health status/QoL as prognostically significant across different cancer types.^22^^,^^23^

The findings of this study have implications for both trialists and clinicians. In clinical trial design, baseline HRQoL scores could be considered as a stratification factor given their prognostic effect to improve efficiency and minimise confounding. However, implementation remains a challenge, especially when several HRQoL scales are prognostic, making it difficult to include multiple stratification factors. Furthermore, the fact that HRQoL data is typically not collected before randomisation poses an additional challenge. Some guidance is needed in this regard. This complementary utility to clinical variables in patient assessment could also encourage HRQoL collection in clinical practice. In conjunction with identified thresholds of clinical importance^38^ for the QLQ-C30, our findings could guide clinicians on which scales should be the focus at the start of follow-up to both improve prognosis and address clinically important impairments. Future research could develop clinical prediction rules based on HRQoL for clinical practice.^39^ Continued follow-up of HRQoL over the course of treatment could also be used as a separate indicator of deterioration. However, respondent fatigue and the resulting poor completion rates of HRQoL measures in trials and clinical practice^40^ may pose a challenge to its effective use.

While our study affirmed the added prognostic value of physical functioning, pain, and appetite loss at baseline on survival, the improvement in predictive capability was smaller than Quinten et al.’s^25^ findings. While the *C* index increase was limited (2.8%), the actual predictive accuracy was higher compared to Quinten et al.’s^25^ findings (0.74 vs. 0.72). Furthermore, the HRs of the HRQoL scales were smaller than those of the clinical and sociodemographic variables. This has been well-documented in numerous prognostic factor analyses^8^^,^^9^^,^^12^ suggesting that clinically significant differences in HRQoL may not always translate to clinically meaningful effects on survival. Ediebah et al.^27^ and Quinten et al.^25^ reported larger HRs when analysing a specific cancer type, whereas our study included multiple cancer types. Also, a global analysis by Quinten et al.^41^ found different prognostic HRQoL scales per cancer type. Post-hoc analyses revealed slight variations in HRs across different cancer types, with some showing increased magnitudes compared to the pooled sample. Additionally, our final model retained more HRQoL scales than Quinten et al.^25^ which did not seem to translate to a noticeable improvement in model performance (gain of 0.003 in *C* index). This small gain in predictive ability compared to increase in complexity should motivate using smaller subsets of HRQoL domains for predictive models.

These findings suggest that imposing a uniform set of prognostic factors across multiple cancer types might dampen the impact of HRQoL, potentially explaining the smaller HRs and less improvement in predictive capability. Post-hoc analyses further supported this, showing variations in the number of scales retained between cohorts defined by metastatic status and the overall sample. Bootstrapping exercises^9^^,^^18^ also showed that, although selection proportions of individual scales were acceptable, model instability was high in terms of the selected sets of HRQoL scales. Hence, though HRQoL may be generally prognostic, the specific facets that hold prognostic value could vary among cancer type. Further research is needed to examine HRQoL's prognostic value in more homogeneous cancer groups.

Our study did not only include patients across several cancer types, but also encompassed diverse disease settings (e.g., metastatic vs. non-metastatic) and varied prior treatment statuses. These factors can influence HRQoL at baseline. In fact, exploration of baseline HRQoL across cancer types has also provided insight into how the scores could highlight differences in symptoms or impairment of certain cancer types. Better scores across both symptom and function scales for melanoma and prostate cancer patients could be explained by the fact that the data for these cancer types in our study mostly came from non-metastatic cancer patients who were more likely to be treatment-naïve. Different types of anti-cancer treatment have been found to contribute to fatigue, insomnia, and chronic pain, and to impair sexual and cognitive functioning.^42^^,^^43^ Previous treatments could then lead to worse self-assessment of baseline HRQoL for these metastatic patients. Furthermore, most patients in our study received traditional cytotoxic cancer treatments. However, the ongoing shift towards targeted/immune therapies, which impact patients' HRQoL differently, raises questions about how this shift affects the prognostic value of HRQoL. Taking this into consideration, future research should investigate the prognostic value of HRQoL and its particularities for specific disease settings such as cancer type, metastasis status and treatment type.

Closer inspection of our findings showed that the HRs for emotional and cognitive functioning scales were in the opposite direction to what we expected; improved survival was associated with lower levels of emotional and cognitive functioning. This reversed effect of emotional functioning was also found in a prognostic factor analysis among glioma patients.^18^ Cognitive functioning, however, showed the expected effect in a prognostic factor analysis among glioblastoma patients.^19^ Notably, in our study, this counterintuitive effect appeared only in the multivariable model, suggesting multicollinearity. This suggests that the covariates were closely related, possibly making the counterintuitive association a modelling artefact. Machingura et al.^44^ showed that the various QLQ-C30 scales are interrelated. Additionally, inherent multicollinearity of the QLQ-C30 was found to contribute to model instability, even with low pairwise correlations.^45^ Future prognostic analyses might use correlation cut-offs below the commonly used 0.8 during model building to lessen multicollinearity among HRQoL scales. Future research could also investigate how the QLQ-C30 summary score (averaging 13 of the 15 scales, excluding global health status and financial difficulty)^46^ serves as a prognostic factor for survival, compared to the individual scales, to mitigate potential multicollinearity. While our study focused on the QLQ-C30 to validate the findings of Quinten et al.,^25^ other PRO tools have also been employed to assess the prognostic value of HRQoL on overall survival.^13^^,^^14^^,^^47^ Validating results from these studies can improve generalizability by establishing the prognostic value of HRQoL, regardless of PRO tool. Finally, the relationship between HRQoL and overall survival could be examined further. This can be done by exploring the added value of longitudinal HRQoL assessments or, similarly, the surrogacy of HRQoL deterioration for survival.^48^

As a pooled analysis and validation study, this study has some limitations. Complete case analysis was used to address missing data. A majority of missingness (17%) was due to missing sociodemographic information. However, these patients did not differ systematically from the rest of the sample in terms of their HRQoL scores or survival status. Harmonization of data from different trials from different databases also limited available useable information. One example was the lack of immediate information of metastasis status in some trials. This necessitated deriving status from several other variables yielding a less informative categorization to standardise information across all the trials.

In conclusion, this study confirms that baseline HRQoL measured using the EORTC QLQ-C30—specifically physical functioning, pain, and appetite loss—is prognostic for survival across several cancer types. Additionally, this study also identified global health status/QoL, dyspnoea, and emotional and cognitive functioning to be prognostic for survival even when accounting for other known prognostic factors.

## Contributors

J. Musoro, M. Pe, C. Coens, E. Basch, Y. Brandberg, F. Cardoso, A. Eggermont, M. Groenvold, J. Ringash, R. Soffietti, M. Taphoorn, W. Van der Graaf, J. Van Meerbeeck, G. Velikova, A. Bottomley, and H. Flechtner were involved in the conceptualization of the study and funding acquisition. J. Musoro, M. Pe, C. Coens, A. Machingura, and L. Lim were involved in defining the methodology. Data acquisition was done by M. Pe, J. Musoro, C. Coens, A. Bottomley, J. Ringash, and D. Tu. Data curation was done by L. Lim, A. Machingura, and M. Taye. Data was assessed and verified by L. Lim and A. Machingura. Analysis was done by L. Lim. J. Musoro, M. Pe, and C. Coens supervised the analysis. L. Lim, J. Musoro, M. Pe, C. Coens, A. Machingura, A. Alanya, F. Martinelli, and S. Antunes were involved in writing the original draft. All authors reviewed and edited the manuscript.

## Data sharing statement

The data used in this study are available for data sharing based on the EORTC data sharing policy that can be found on the following link: policy on data sharing.

## Declaration of interests

E. Basch has received payments as a scientific advisor for Navigating Cancer, AstraZeneca, Resilience, and Verily. A Eggermont receives honoraria from BMS, Merck, and MSD. F. Cardoso has received payments for consulting fees from Amgen, Astellas/Medivation, AstraZeneca, Celgene, Daiichi-Sankyo, Eisai, GE Oncology, Genentech, Gilead, GlaxoSmithKline, Iqvia, Macrogenics, Medscape, Merck-Sharp, Merus BV, Mylan, Mundipharma, Novartis, Pfizer, Pierre-Fabre, prIME Oncology, Roche, Sanofi, Samsung Bioepis, Seagen, Teva, and Touchime. W.T.*A. van* der Graaf received an institutional grant from Eli Lilly, participated on an advisory board for Agenus and PTC Therapeutics, and is the President of EORTC and an European Cancer Organization board member. J.A.F. Koekkoek received payments for consulting fees from Fagron and grants paid to the institution from the EORTC Quality of Life Group, EORTC Brain Tumour Group, ZonMw, Team Westland, and the KWF Dutch Cancer Society. G. Velikova received payment from the University of Leeds for her work on this research; grants paid to the institution from the NIHR programme grant, Pfizer, and Yorkshire Cancer Research; payments for consulting fees from Pfizer, Roche, and Seagen; honoraria from Pfizer, Roche, Novartis, Eisai, and Sanofi; support for attending meetings from Pfizer and Roche; participated in a Data Safety Monitoring Board or Advisory Board for Roche, Seagen, and AstraZeneca; and has a leadership or fiduciary role on the EORTC Board of Directors and the NCRI Living with and beyond cancer group. A. Bottomley owns an independent private QOL consulting company that has clients in the pharmaceutical industry. All other authors declare no competing interests.

## Appendix ASupplementary data

Appendix Figs. A1–A11 and Tables A1–A3
