# Supplementary material for: Prognostic value of baseline EORTC QLQ-C30 scores for overall survival across 46 clinical trials covering 17 cancer types: a validation study
Source: eClinicalMedicine. 2025 Mar 21;82:103153. doi: 10.1016/j.eclinm.2025.103153 (PMC11976232; doi:10.1016/j.eclinm.2025.103153)
Supplement: Appendix Figs. A1–A11 and Tables A1–A3 [file mmc1.docx]

# APPENDIX

**Collaborator Groups:**

- the EORTC Quality of Life Group
- the EORTC Brain Tumour Group
- the EORTC Breast Cancer Group
- the EORTC Melanoma Group
- the EORTC Lung Cancer Group
- the EORTC Soft Tissue and Bone Sarcoma Group
- the EORTC Radiation Oncology Group
- the EORTC Lymphoma Group
- the EORTC Gastrointestinal Tract Cancer Group
- the EORTC Head and Neck Cancer Group
- the EORTC Genito-Urinary Cancer Group
- the EORTC Gynaecological Cancer Group

Figure A1 shows the flowchart of the bootstrap procedure. The model building procedure above (*step 1 & 2*) was repeated 200 times, each time using a bootstrap data set (data_b) obtained from data_o by sampling with replacement.^1^ For each bootstrap run, the *C* Index for the apparent (app_b) and test (test_b) performance were obtained by assessing the final bootstrap model (fmodel_b) on data_b and data_o respectively. Optimism was computed by subtracting test_b from app_b. The optimism-corrected C Index was computed as the difference of the apparent *C* Index (app_o) from *step 2* and the mean of the 200 optimism estimates form the bootstrap procedure. The selection frequency of all variables was monitored for the various bootstrap final models.

**Figure A1. Flowchart of Bootstrap Process for Obtaining Corrected C-Indices
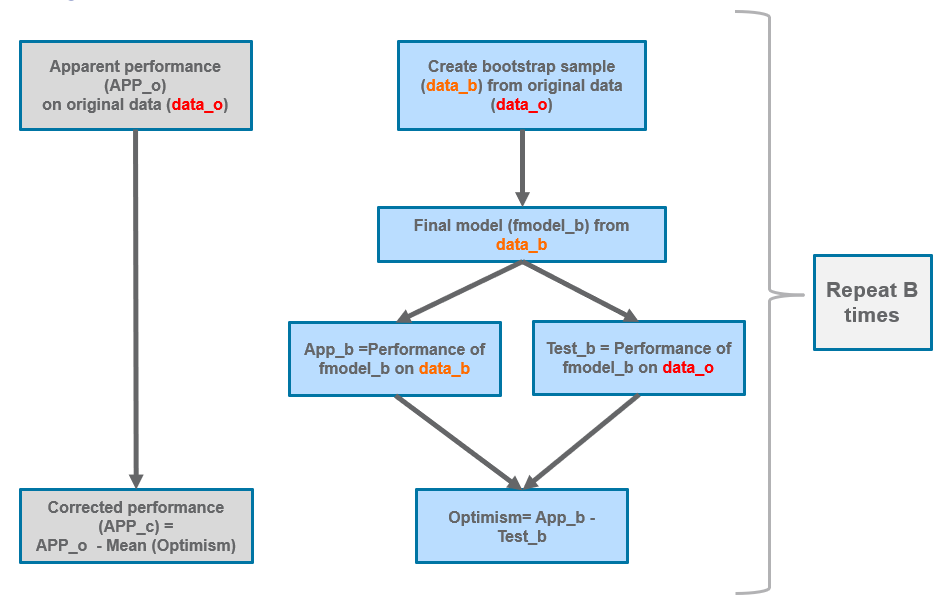
**

**Figure A2. Sample Size Flowchart**

**Figure A3. Frequency of selection for Clinical and HRQoL Variables across 200 Bootstraps**

13,309 patients

17 cancer types

2,901 patients excluded

- Missing sociodemographic data

16,210 patients with complete HRQoL and survival data

46 closed RCTs

16,863 patients

653 patients excluded

- 624 missing baseline HRQoL score
- 29 missing survival data

**(Top: Model QX1, Bottom: Model QX2)**

Model QX1, final model without restriction in model selection; Model QX2, final model forcing sociodemographic and clinical variables into the model; WHO PS, WHO performance status; PF, Physical functioning; RF, role functioning; EF, emotional functioning; CF, cognitive functioning; SF, social functioning; QL, global health status/quality of life; FA, fatigue; NV, nausea/ vomiting; PA, pain; DY, dyspnoea; SL, insomnia; AP, appetite loss; CO, constipation; DI, diarrhoea; FI, financial problems

**Table A1. Overview of trials included in this study**

| **Study Number** | **EudraCT/NCT Number** | **Title** |
| --- | --- | --- |
| EORTC-22993-08993 | NCT00016211 | Prophylactic Cranial Irradiation in Extensive Disease Small Cell Lung Cancer |
| EORTC-08092 | 2010-018566-23 | Double Blind Randomized Phase III Study of Maintenance Pazopanib Versus Placebo in NSCLC Patients Non Progressive After First Line Chemotherapy |
| EORTC-08971 | NCT00003279 | The SILVA Study: Survival in an International Phase III Prospective Randomized LD Small Cell Lung Cancer Vaccination Study with Adjuvant BEC2 and BCG |
| EORTC-18071 | 2007-001974-10 | Adjuvant Immunotherapy with Anti-CTLA-4 Monoclonal Antibody (Ipilimumab) Versus Placebo After Complete Resection of High Risk Stage III Melanoma: A Randomized, Double-blind Phase 3 Trial of the EORTC Melanoma Group |
| EORTC-20012 | 2004-001558-10 | BEACOPP (4 cycles escalated + 4 cycles baseline) Versus  ABVD (8 cycles) in Stage III & IV Hodgkin’s Lymphoma |
| EORTC-30974 | NCT00003941 | A Randomized Phase III Study of Sequential High-Dose Cisplatinum/Etoposide/Ifosfamide Plus Stem Cell Support Versus BEP in Patients with Poor Prognosis Germ Cell Cancer |
| EORTC-30983 | 2004-000731-28 | A Randomised Phase II/III Study of Taxol-BEP Versus BEP in Patients with Intermediate Prognosis Germ Cell Cancer |
| EORTC-22991 | NCT00021450 | Three Dimensional Conformal Radiotherapy / Intensity Modulated Radiotherapy Alone Vs Three Dimensional Conformal Therapy / Intensity Modulated Radiotherapy Plus Adjuvant Hormonal Therapy In Localized T1b-c, T2a, N0, M0 Prostatic Carcinoma. A Phase III Randomized Study |
| EORTC-10981-22023 | NCT00014612 | After Mapping of The Axilla: Radiotherapy Or Surgery |
| EORTC-22033-26033 | 2004-002714-11 | Primary chemotherapy with temozolomide vs. radiotherapy in patients with low grade gliomas after stratification for genetic 1p loss: a phase III study |
| EORTC-30004 | NCT00042887 | Chemoresection With 4 Weekly Intravesical Instillations Of Mitomycin C Versus Transurethral Resection (TUR) Followed By One Single Immediate Instillation Of Mitomycin C In Single, Small, Papillary Stage Ta, T1 Bladder Tumors: A Prospective Randomized Phase III Trial |
| EORTC-30986 | NCT00014274 | Randomized Phase II/III Study Assessing Gemcitabine/Carboplatin And Methotrexate/Carboplatin/Vinblastine In Previously Untreated Patients With Advanced Urothelial Cancer Ineligible For Cisplatin Based Chemotherapy |
| EORTC-40954 | NCT00004099 | Randomized Phase III Study of Preoperative Chemotherapy Followed by Surgery Versus Surgery Alone in Locally Advanced Gastric Cancer (cT3 and cT4NxM0) |
| EORTC-40013-22012 | 2005-000933-39 | Randomized Phase II/III Study Comparing Gemcitabine Followed by Gemcitabine Plus Concomitant Radiation (50.4 Gy) Versus Control After Curative Pancreaticoduodenectomy for Pancreatic Head Cancer |
| EORTC-55041 | 2004-004333-34 | A Randomized, Multicenter, Phase III Study of Erlotinib Versus Observation in Patients With no Evidence of Disease Progression After First Line, Platinum-Based Chemotherapy For High-Risk Ovarian Epithelial, Primary Peritoneal, or Fallopian Tube Cancer |
| EORTC-55955 | NCT00002895 | A Randomised Trial in Relapsed Ovarian Cancer: Early Treatment Based on CA 125 Levels Alone Vs. Delayed Treatment Based On Conventional Clinical Indicators |
| EORTC-55971 | NCT00003636 | A Randomized Phase III Study Comparing Upfront Debulking Surgery Versus Neo-Adjuvant Chemotherapy in Patients With Stage IIIC or IV Epithelial Ovarian Carcinoma |
| EORTC-55984 | NCT00052312 | Randomized Trial Of Adriamycin (A) Cisplatin (P) Chemotherapy Versus Paclitaxel (T) Adriamycin (A) And Cisplatin (P) In Patients With Metastatic/Relapsed Or Locally Advanced Inoperable Endometrial Cancer |
| EORTC-62072 | 2008-001307-33 | A Randomized Double Blind Phase III Trial of Pazopanib Versus Placebo in Patients With Soft Tissue Sarcoma Whose Disease Has Progressed During or Following Prior Therapy |
| EORTC-62091 | 2009-014889-26 | TRUSTS: A Phase IIB/III Multicenter Study Comparing the Efficacy of TRabectedin Administered as a 3-Hour or 24-Hour Infusion to Doxorubicin in Patients With Advanced or Metastatic Untreated Soft Tissue Sarcoma |
| EORTC-40004 | NCT00043004 | CLOCC Trial (Chemotherapy + Local Ablation Versus Chemotherapy) Randomized Phase II Study Of Local Treatment Of Liver Metastases By Radiofrequency Combined With Chemotherapy Versus Chemotherapy Alone In Patients With Unresectable Colorectal Liver Metastases |
| EORTC-22011-40014 | 2004-000329-31 | Continuous Fluorouracil Plus Mitomycin C Versus Mitomycin C Plus Cisplatin As Chemotherapy Combination In Combined Radiochemotherapy For Locally Advanced Anal Cancer. A Phase II-III Study |
| EORTC-24971 | NCT00003888 | A Randomized Phase III Multicenter Trial of Neoadjuvant Docetaxel (Taxotere) Plus Cisplatin Plus 5-Fluorouracil Versus Neoadjuvant Cisplatin Plus 5-Fluorouracil in Patients With Locally Advanced Inoperable Squamous Cell Carcinoma of the Head and Neck |
| CAN-NCIC-BR20 | NCT00066313 | A Phase II Study Of ZD6474 Or Placebo In Small Cell Lung Cancer Patients Who Have Complete Or Partial Response To Induction Chemotherapy +/- Radiation Therapy |
| BR.21 | NCT00036647 | A Randomized Placebo Controlled Study of OSI-774 (Erlotinib HCl, Tarceva[TM]) in Patients With Incurable Stage IIIB/IV Non-small Cell Lung Cancer Who Have Failed Standard Therapy for Advanced or Metastatic Disease |
| CAN-NCIC-BR24 | NCT00245154 | A Phase II/III Double Blind Randomized Trial of AZD2171 Versus Placebo in Patients Receiving Paclitaxel/Carboplatin Chemotherapy for the Treatment of Advanced or Metastatic Non-Small Cell Lung Cancer |
| CAN-NCIC-BR25 | NCT00346320 | A Phase II Study of Accelerated Hypofractionated 3-Dimensional Conformal Radiotherapy (3DCRT) For Inoperable Stage I/II Non-Small Cell Lung Cancer (NSCLC) |
| CAN-NCIC-BR26 | NCT01000025 | A Double Blind Placebo Controlled Randomized Trial of PF-804 in Patients With Incurable Stage IIIB/IV Non-Small Cell Lung Cancer After Failure of Standard Therapy for Advanced or Metastatic Disease |
| CAN-NCIC-BR29 | NCT00795340 | A Double Blind Randomized Trial of Cediranib Versus Placebo in Patients Receiving Paclitaxel/Carboplatin Chemotherapy for the Treatment of Advanced or Metastatic Non-Small Cell Lung Cancer |
| CAN-NCIC-MA21 | NCT00014222 | A Phase III Adjuvant Trial Of Sequenced EC + Filgrastim + Epoetin Alfa Followed By Paclitaxel Versus Sequenced AC Followed By Paclitaxel Versus CEF As Therapy For Premenopausal Women And Early Postmenopausal Women Who Have Had Potentially Curative Surgery For Node Positive Or High Risk Node Negative Breast Cancer |
| CAN-NCIC-MA31 | NCT00667251 | A Randomized, Open-Label, Phase III Study of Taxane Based Chemotherapy With Lapatinib or Trastuzumab as First-Line Therapy for Women With HER2/Neu Positive Metastatic Breast Cancer |
| CAN-NCIC-CE6 | NCT00482677 | A Randomized Phase III Study of Temozolomide and Short-Course Radiation Versus Short-Course Radiation Alone In The Treatment of Newly Diagnosed Glioblastoma Multiforme in Elderly Patients |
| CAN-NCIC-PA3 | NCT00026338 | A Randomized Placebo Controlled Study Of OSI-774 (TARCEVA) Plus Gemcitabine In Patients With Locally Advanced, Unresectable Or Metastatic Pancreatic Cancer |
| CAN-NCIC-OV16 | NCT00028743 | A Phase III Study of Cisplatin Plus Topotecan Followed by Paclitaxel Plus Carboplatin Versus Paclitaxel Plus Carboplatin as First Line Chemotherapy in Women With Newly Diagnosed Advanced Epithelial Ovarian Cancer |
| CAN-NCIC-OV21 | NCT00993655 | A Phase II Study of Intraperitoneal (IP) Plus Intravenous (IV) Chemotherapy Versus IV Carboplatin Plus Paclitaxel in Patients With Epithelial Ovarian Cancer Optimally Debulked at Surgery Following Neoadjuvant Intravenous Chemotherapy |
| CAN-NCIC-CO17 | NCT00079066 | A Phase III Randomized Study of Cetuximab (Erbitux™, C225) and Best Supportive Care Versus Best Supportive Care in Patients With Pretreated Metastatic Epidermal Growth Factor Receptor (EGFR)-Positive Colorectal Carcinoma |
| CAN-NCIC-CO20 | NCT00640471 | A Phase III Randomized Study of Brivanib Alaninate (BMS-582664) in Combination With Cetuximab (Erbitux®) Versus Placebo in Combination With Cetuximab (Erbitux®) in Patients With K-RAS Wild Type Tumors Previously Treated With Combination Chemotherapy for Metastatic Colorectal Carcinoma |
| CO23 | NCT01830621 | A Phase III Randomized Study of BBI608 and Best Supportive Care Versus Placebo and Best Supportive Care in Patients With Pretreated Advanced Colorectal Carcinoma |
| I210 | NCT01622543 | A Randomized Phase II Study of Reolysin in Combination With FOLFOX6 and Bevacizumab or FOLFOX6 and Bevacizumab Alone in Patients With Metastatic Colorectal Cancer. |
| CAN-NCIC-JMY10 | NCT00049673 | A Randomized Phase III Study Of Thalidomide And Prednisone As Maintenance Therapy Following Autologous Stem Cell Transplant in Patients With Multiple Myeloma |
| CAN-NCIC-SC19 | NCT00016380 | A Randomized Phase III Double-Blind Study Of Ondansetron And Dexamethasone Versus Ondansetron And Placebo In The Prophylaxis Of Radiation-Induced Emesis |
| CAN-NCIC-SC20 | NCT00080912 | A Phase III International Randomized Trial Of Single Versus Multiple Fractions For Re-Irradiation Of Painful Bone Metastases |
| RC0639 | NCT00436566 | Phase II Study of Cardiac Safety and Tolerability of an Adjuvant Chemotherapy Plus Trastuzumab With Lapatinib in Patients With Resected HER2 + Breast Cancer |
| 954651 |  | A Phase II Trial of a Seven Day Regimen of Oral 776C85 and Oral 5-Flourouracil (5-FU) in Untreated Patients with Unresectable or Metastatic Colorectal Cancer* |
| A021202 | NCT01841736 | Prospective Randomized Phase II Trial of Pazopanib (NSC #737754) Versus Placebo in Patients With Progressive Carcinoid Tumors |
| MC1345 | NCT02265341 | Pilot Study of Ponatinib in Biliary Cancer Patients With FGFR2 Fusions |

* Study was conducted by the Mayo Clinic

**Table A2. Patient Baseline Characteristics by Cancer Type**

| **Patient Characteristic** | **Total**  **(N=16210)** | **Anal**  **(N=60)** | **Bladder**  **(N=228)** | **Brain**  **(N=822)** | **Breast**  **(N=3466)** | **Colorectal**  **(N=1693)** | | **Endometrial**  **(N=92)** | | **Gastric**  **(N=324)** | | **Head and Neck**  **(N=330)** | | **Lung**  **(N=2732)** | |  |
| --- | --- | --- | --- | --- | --- | --- | --- | --- | --- | --- | --- | --- | --- | --- | --- | --- |
|  | **N (%)** | **N (%)** | **N (%)** | **N (%)** | **N (%)** | **N (%)** | | **N (%)** | | **N (%)** | | **N (%)** | | **N (%)** | |  |
| **Age** |  |  |  |  |  | |  | |  | |  | |  | |  | |
| Median | 58·0 | 56·5 | 70 | 69 | 50·7 | | 63·3 | | 62 | | 60 | | 53 | | 62 | |
| Range | 16–93 | 41·0-75·0 | 25·0-87·0 | 18·0-90·0 | 22·7-87·1 | | 27·0-88·1 | | 34·0-75·0 | | 26·0-89·0 | | 30·0-70·0 | | 23·0-90·2 | |
| ≤60 years | 9130 (56·3) | 37 (61·7) | 47 (20·6) | 303 (36·9) | 2856 (82·4) | | 646 (38·2) | | 38 (41·3) | | 164 (50·6) | | 266 (80·6) | | 1197 (43·8) | |
| >60 years | 7080 (43·7) | 23 (38·3) | 181 (79·4) | 519 (63·1) | 610 (17·6) | | 1047 (61·8) | | 54 (58·7) | | 160 (49·4) | | 64 (19·4) | | 1535 (56·2) | |
| **Sex** |  |  |  |  |  | |  | |  | |  | |  | |  | |
| Male | 6647 (41·0) | 15 (25·0) | 174 (76·3) | 491 (59·7) | 0 (0·0) | | 1094 (64·6) | | 0 (0·0) | | 176 (54·3) | | 297 (90·0) | | 1567 (57·4) | |
| Female | 9563 (59·0) | 45 (75·0) | 54 (23·7) | 331 (40·3) | 3466 (100·0) | | 599 (35·4) | | 92 (100·0) | | 148 (45·7) | | 33 (10·0) | | 1165 (42·6) | |
| **WHO Performance Status** |  |  |  |  |  | |  | |  | |  | |  | |  | |
| Active (Performance Status = 0) | 7211 (44·5) | 49 (81·7) | 61 (26·8) | 334 (40·6) | 1708 (49·3) | | 546 (32·3) | | 41 (44·6) | | 114 (35·2) | | 167 (50·6) | | 629 (23·0) | |
| Restricted | 7558 (46·6) | 11 (18·3) | 167 (73·2) | 488 (59·4) | 489 (14·1) | | 1147 (67·7) | | 51 (55·4) | | 70 (21·6) | | 163 (49·4) | | 2102 (76·9) | |
| Performance Status = 1 | 6317 (39·0) | 11 (18·3) | 82 (36·0) | 363 (44·2) | 466 (13·4) | | 953 (56·3) | | 47 (51·1) | | 67 (20·7) | | 0 (0·0) | | 1663 (60·9) | |
| Performance Status = 2 | 1157 (7·1) | 0 (0·0) | 85 (37·3) | 125 (15·2) | 23 (0·7) | | 194 (11·5) | | 4 (4·3) | | 3 (0·9) | | 0 (0·0) | | 362 (13·3) | |
| Performance Status = 3 | 84 (0·5) | 0 (0·0) | 0 (0·0) | 0 (0·0) | 0 (0·0) | | 0 (0·0) | | 0 (0·0) | | 0 (0·0) | | 0 (0·0) | | 77 (2·8) | |
| Missing | 1441 (8·9) | 0 (0·0) | 0 (0·0) | 0 (0·0) | 1269 (36·6) | | 0 (0·0) | | 0 (0·0) | | 140 (43·2) | | 0 (0·0) | | 1 (0·0) | |
| **Metastasis Status** |  |  |  |  |  | |  | |  | |  | |  | |  | |
| Metastatic | 5709 (35·2) | 0 (0·0) | 194 (85·1) | 0 (0·0) | 618 (17·8) | | 1693 (100·0) | | 85 (92·4) | | 0 (0·0) | | 0 (0·0) | | 1636 (59·9) | |
| Non-metastatic | 8837 (54·6) | 60 (100·0) | 34 (14·9) | 329 (40·0) | 2804 (80·9) | | 0 (0·0) | | 7 (7·6) | | 109 (33·6) | | 330 (100·0) | | 995 (36·4) | |
| Missing | 1664 (10·2) | 0 (0·0) | 0 (0·0) | 493 (60·0) | 44 (1·3) | | 0 (0·0) | | 0 (0·0) | | 215 (66·4) | | 0 (0·0) | | 101 (3·7) | |

**Table A2. Patient Baseline Characteristics by Cancer Type (cont.)**

| **Patient Characteristic** | **Lymphoma**  **(N=348)** | **Melanoma**  **(N=861)** | **Mixed population**  **(N=811)** | **Multiple myeloma**  **(N=318)** | **Pancreatic**  **(N=516)** | **Prostate**  **(N=645)** | **Ovarian**  **(N=2269)** | **Soft tissue sarcoma**  **(N=425)** | **Testicular**  **(N=270)** |
| --- | --- | --- | --- | --- | --- | --- | --- | --- | --- |
|  | **N (%)** | **N (%)** | **N (%)** | **N (%)** | **N (%)** | **N (%)** | **N (%)** | **N (%)** | **N (%)** |
| **Age** |  |  |  |  |  |  |  |  |  |
| Median | 34 | 51 | 61·6 | 57·8 | 63 | 70 | 59 | 56 | 28 |
| Range | 16·0-60·0 | 18·0-84·0 | 16·5-93·0 | 33·2-74·5 | 32·0-92·4 | 49·0-80·0 | 19·0-86·0 | 18·0-84·0 | 16·0-50·0 |
| ≤60 years | 348 (100·0) | 640 (74·3) | 370 (45·6) | 197 (61·9) | 203 (39·3) | 49 (7·6) | 1228 (54·1) | 271 (63·8) | 270 (100·0) |
| >60 years | 0 (0·0) | 221 (25·7) | 441 (54·4) | 121 (38·1) | 313 (60·7) | 596 (92·4) | 1041 (45·9) | 154 (36·2) | 0 (0·0) |
| **Sex** |  |  |  |  |  |  |  |  |  |
| Male | 260 (74·7) | 528 (61·3) | 460 (56·7) | 211 (66·4) | 285 (55·2) | 645 (100·0) | 0 (0·0) | 174 (40·9) | 270 (100·0) |
| Female | 88 (25·3) | 333 (38·7) | 351 (43·3) | 107 (33·6) | 231 (44·8) | 0 (0·0) | 2269 (100·0) | 251 (59·1) | 0 (0·0) |
| **WHO Performance Status** |  |  |  |  |  |  |  |  |  |
| Active (Performance Status = 0) | 117 (33·6) | 806 (93·6) | 273 (33·7) | 118 (37·1) | 160 (31·0) | 565 (87·6) | 1162 (51·2) | 207 (48·7) | 154 (57·0) |
| Restricted | 231 (66·4) | 55 (6·4) | 508 (62·6) | 200 (62·9) | 355 (68·8) | 80 (12·4) | 1107 (48·8) | 218 (51·3) | 116 (43·0) |
| Performance Status = 1 | 169 (48·6) | 55 (6·4) | 390 (48·1) | 187 (58·8) | 273 (52·9) | 79 (12·2) | 1039 (45·8) | 218 (51·3) | 92 (34·1) |
| Performance Status = 2 | 61 (17·5) | 0 (0·0) | 116 (14·3) | 13 (4·1) | 82 (15·9) | 1 (0·2) | 68 (3·0) | 0 (0·0) | 20 (7·4) |
| Performance Status = 3 | 1 (0·3) | 0 (0·0) | 2 (0·2) | 0 (0·0) | 0 (0·0) | 0 (0·0) | 0 (0·0) | 0 (0·0) | 4 (1·5) |
| Missing | 0 (0·0) | 0 (0·0) | 30 (3·7) | 0 (0·0) | 1 (0·2) | 0 (0·0) | 0 (0·0) | 0 (0·0) | 0 (0·0) |
| **Metastasis Status** |  |  |  |  |  |  |  |  |  |
| Metastatic | 257 (73·9) | 0 (0·0) | 0 (0·0) | 0 (0·0) | 269 (52·1) | 1 (0·2) | 460 (20·3) | 424 (99·8) | 72 (26·7) |
| Non-metastatic | 91 (26·1) | 861 (100·0) | 0 (0·0) | 318 (100·0) | 247 (47·9) | 644 (99·8) | 1809 (79·7) | 1 (0·2) | 198 (73·3) |
| Missing | 0 (0·0) | 0 (0·0) | 811 (100·0) | 0 (0·0) | 0 (0·0) | 0 (0·0) | 0 (0·0) | 0 (0·0) | 0 (0·0) |

|  | Metastatic | | Non-metastatic | |
| --- | --- | --- | --- | --- |
|  | Univariable Cox Models | Cox Model for Sociodemographic, Clinical, and HRQoL Data  (CPHM_QX2_) | Univariable Cox Models | Cox Model for Sociodemographic, Clinical, and HRQoL Data  (CPHM_QX2_) |
|  | Hazard ratio (95% CI) | Hazard ratio (95% CI) | Hazard ratio (95% CI) | Hazard ratio (95% CI) |
| Sociodemographic and clinical variables* |  |  |  |  |
| Sex (Female vs. Male) | 0·928 (0·866, 0·995) | 0·905 (0·843, 0·971) | 0·833 (0·756, 0·918) | 0·808 (0·733, 0·891) |
| Age (> 60 vs. ≤ 60) | 1·085 (1·017, 1·158) | 0·951 (0·890, 1·015) | 1·196 (1·109, 1·291) | 0·859 (0·796, 0·928) |
| WHO PS (Restricted vs. Active) | 1·644 (1·531, 1·765) | 1·261 (1·168, 1·362) | 1·314 (1·219, 1·416) | 1·167 (1·078, 1·263) |
| QLQ-C30 HRQoL subscales^†^‡ |  |  |  |  |
| Physical Functioning | 0·866 (0·853,0·878) | 0.951 (0.929,0.973) | 0·917 (0·902,0·933) | 0·960 (0·937,0·983) |
| Pain | 1·091 (1·080,1·103) | 1.021 (1.006,1.035) | 1·050 (1·036,1·063) |  |
| Appetite loss | 1·096 (1·085,1·107) | 1.043 (1.031,1.056) | 1·055 (1·043,1·068) | 1·030 (1·016,1·043) |
| Global health status / QoL | 0·883 (0·871,0·895) | 0.960 (0.942,0.979) | 0·937 (0·923,0·952) |  |
| Dyspnoea | 1·068 (1·057,1·080) |  | 1·046 (1·032,1·061) |  |
| Emotional Functioning | 0·963 (0·950,0·977) | 1.039 (1.021,1.057) | 0·978 (0·964,0·993) |  |
| Cognitive Functioning | 0·944 (0·930,0·959) | 1.025 (1.006,1.044) | 0·984 (0·968,1·000) |  |
| Role Functioning | 0·914 (0·905,0·923) |  | 0·951 (0·940,0·961) | 0·982 (0·967,0·998) |
| Social Functioning | 0·930 (0·920,0·940) |  | 0·962 (0·950,0·973) |  |
| Fatigue | 1·129 (1·115,1·142) | 1.026 (1.004,1.048) | 1·057 (1·043,1·071) |  |
| Nausea/Vomiting | 1·139 (1·122,1·156) | 1.026 (1.005,1.049) | 1·056 (1·037,1·076) |  |
| Insomnia | 1·033 (1·022,1·043) |  | 1·017 (1·006,1·029) |  |
| Constipation | 1·046 (1·035,1·058) |  | 1·023 (1·010,1·037) |  |
| Diarrhoea | 1·012 (0·995,1·028) |  | 1·017 (1·000,1·035) |  |
| Financial Problems | 1·025 (1·014,1·036) |  | 1·021 (1·009,1·033) |  |

**Table A3. Univariable and Multivariable Analysis of Clinical and HRQoL Variables by Metastasis Status**

*Reference categories are Female, >60, Restricted, and Yes

†HRs are computed reflecting a 10-pt increase in the scale score

‡ Higher functioning and lower symptom scale scores indicate better HRQoL

**Figure A4.** **Forest Plot of Hazard Ratios of Physical Functioning**


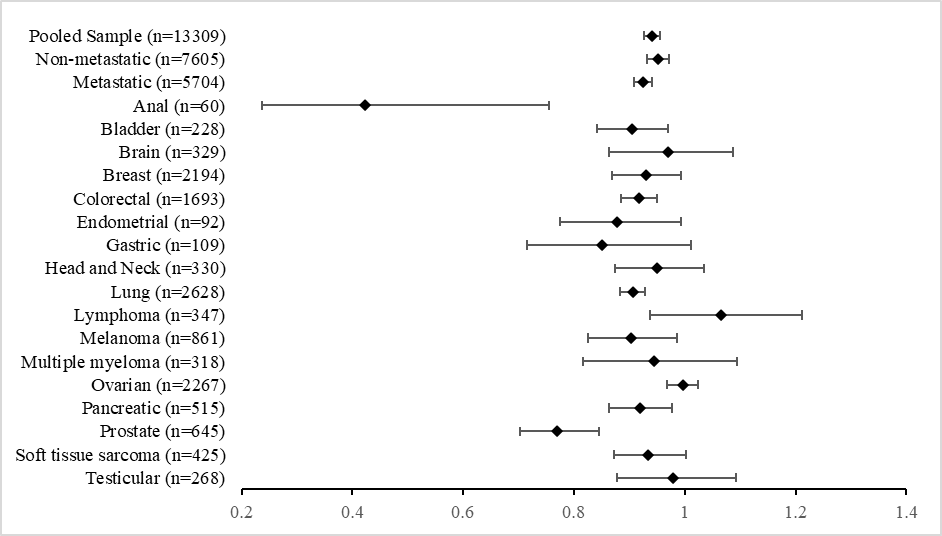


**Figure A5.** **Forest Plot of Hazard Ratios of Pain**


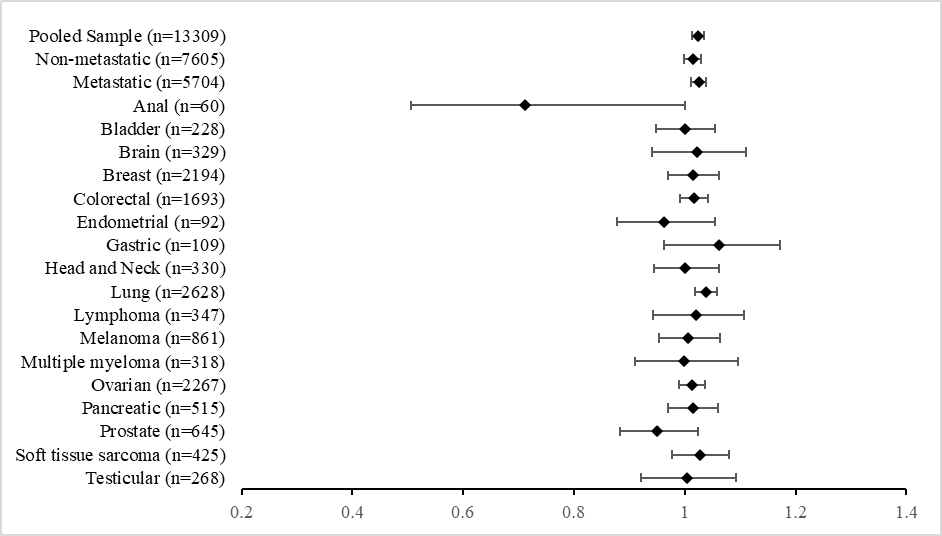


**Figure A6.** **Forest Plot of Hazard Ratios of Appetite Loss**


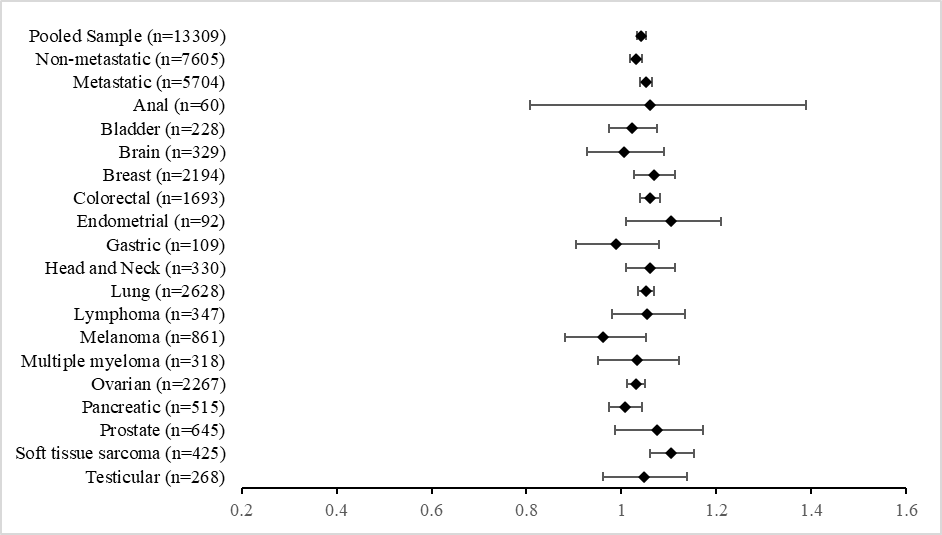


**Figure A7. Kaplan-Meier Curves of Patients with Good Prognosis by Emotional Functioning**


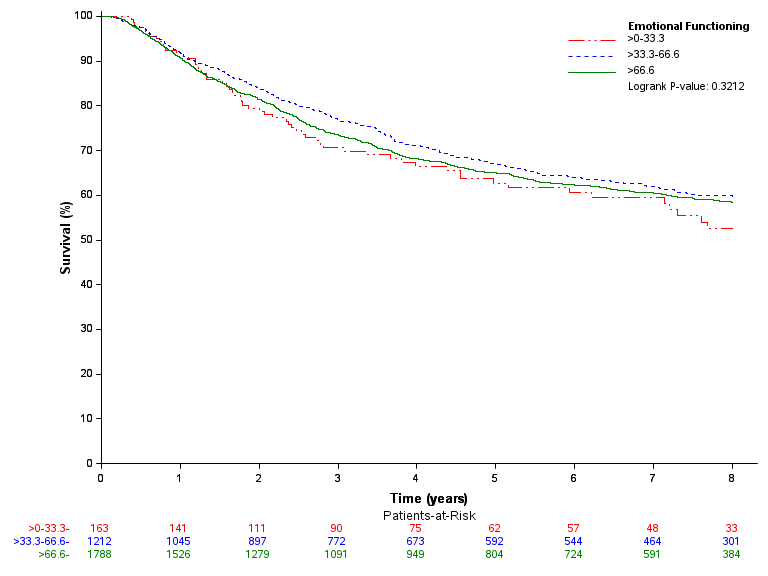


Higher scores indicate better HRQoL

**Figure A8.** **Kaplan-Meier Curves of Patients with Good Prognosis by Cognitive Functioning**


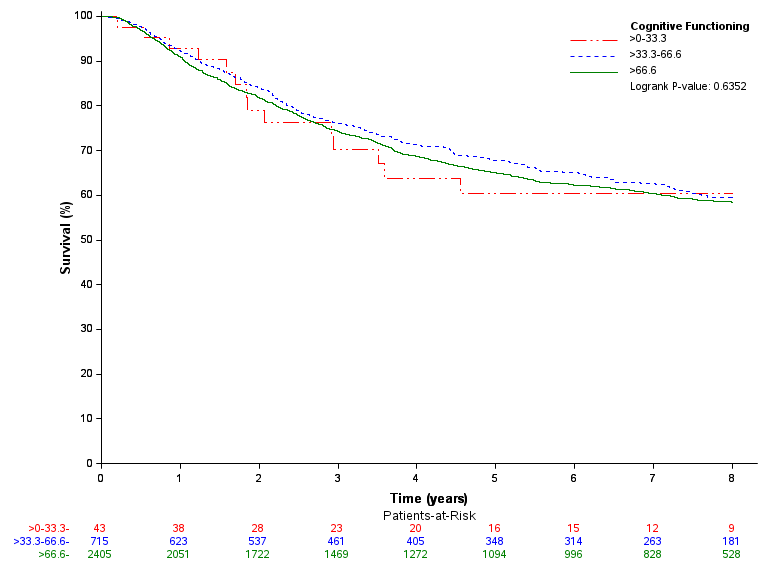


Higher scores indicate better HRQoL

**Figure A9.** **Kaplan-Meier Curves of Patients with Good Prognosis by Pain**


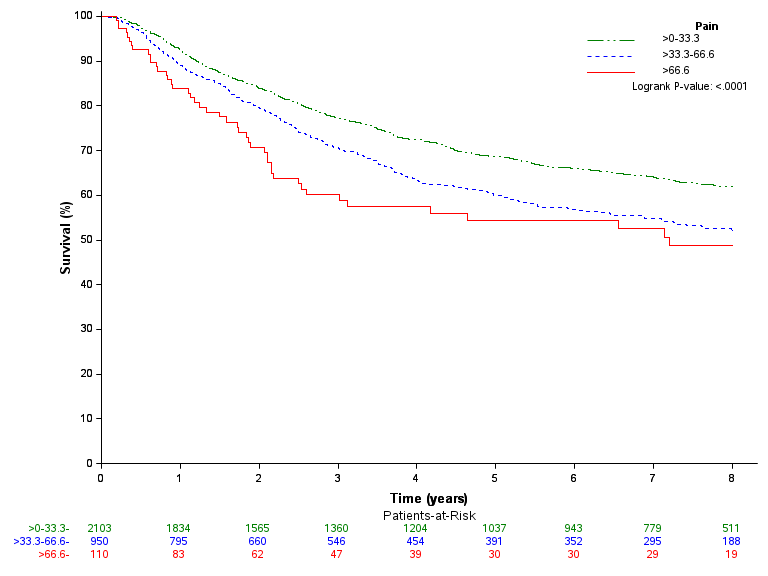


Lower scores indicate better HRQoL

**Figure A10.** **Kaplan-Meier Curves of Patients with Good Prognosis by Dyspnoea**


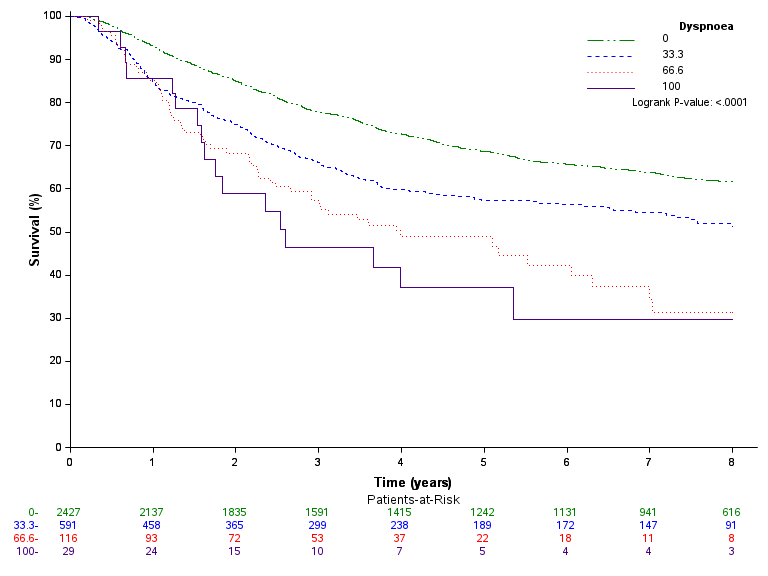


Lower scores indicate better HRQoL

**Figure A11.** **Kaplan-Meier Curves of Patients with Good Prognosis by Appetite Loss**


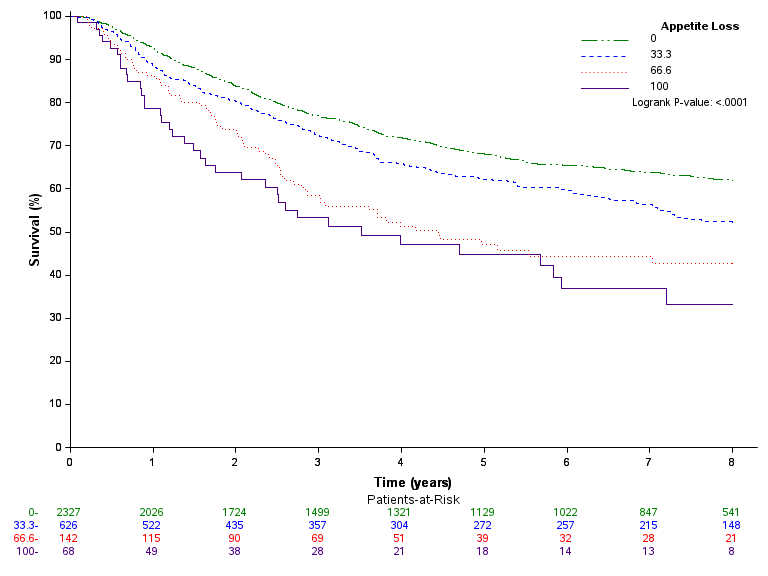


Lower scores indicate better HRQoL

**8. APPENDIX REFERENCES**

1 Sauerbrei W, Schumacher M. A bootstrap resampling procedure for model building:

application to the Cox regression model. *Stat Med* 1992; **11**: 2093–109.
